# Improved Integration Time Estimation of Endogenous Retroviruses with Phylogenetic Data

**DOI:** 10.1371/journal.pone.0014745

**Published:** 2011-03-04

**Authors:** Hugo Martins, Palle Villesen

**Affiliations:** 1 Bioinformatics Research Center, University of Aarhus, Aarhus, Denmark; 2 Computational Biology PhD Program, Instituto Gulbenkian de Ciência, Oeiras, Portugal; Institute of Infectious Disease and Molecular Medicine, South Africa

## Abstract

**Background:**

Endogenous retroviruses (ERVs) are genetic fossils of ancient retroviral integrations that remain in the genome of many organisms. Most loci are rendered non-functional by mutations, but several intact retroviral genes are known in mammalian genomes. Some have been adopted by the host species, while the beneficial roles of others remain unclear. Besides the obvious possible immunogenic impact from transcribing intact viral genes, endogenous retroviruses have also become an interesting and useful tool to study phylogenetic relationships. The determination of the integration time of these viruses has been based upon the assumption that both 5′ and 3′ Long Terminal Repeats (LTRs) sequences are identical at the time of integration, but evolve separately afterwards. Similar approaches have been using either a constant evolutionary rate or a range of rates for these viral loci, and only single species data. Here we show the advantages of using different approaches.

**Results:**

We show that there are strong advantages in using multiple species data and state-of-the-art phylogenetic analysis. We incorporate both simple phylogenetic information and Monte Carlo Markov Chain (MCMC) methods to date the integrations of these viruses based on a relaxed molecular clock approach over a Bayesian phylogeny model and applied them to several selected ERV sequences in primates. These methods treat each ERV locus as having a distinct evolutionary rate for each LTR, and make use of consensual speciation time intervals between primates to calibrate the relaxed molecular clocks.

**Conclusions:**

The use of a fixed rate produces results that vary considerably with ERV family and the actual evolutionary rate of the sequence, and should be avoided whenever multi-species phylogenetic data are available. For genome-wide studies, the simple phylogenetic approach constitutes a better alternative, while still being computationally feasible.

## Introduction

Retroviral infections have been a constant on animal life for millions of years. Occasionally, some of these genetic parasites integrate into the germline as endogenous retroviruses (ERVs). Genetic footprints such as these constitute up to 8% of the human genome [1]. Many of these ERVs lay now dormant, after millions of years of genetic change, whereas some still retain protein coding capability and play roles in the host organism that range from adhesion promotion [2], [3], [4] to immune response modulation [5], while also being implied in diseases such as multiple sclerosis [6], [7] and correlated with certain types of cancer [8].

Being originated from their extant counterparts, ERVs share the same genetic structure and organization. There are three major classes of ERVs – class I ERVs are similar to gammaretroviruses, class II ERVs are closer to beta and alpharetroviruses whereas class III ERVs are more related to spumaviruses [9]. At the genetic level, identifiable common structures such as the 5′ LTR, PBS, Gag, Pro, Pol, Env, PPT and 3′ LTR may or may not be present in an ERV locus [1], [10], [11]. The natural degeneracy of an ERV locus with neutral substitution rate and a divergence limit for nucleotide sequence recognition results in an upper limit for retroviral age that can be detected. Currently, retroviral sequences older than 250 million years cannot be found in today's genomes [12], although ERVs that are evolutionarily selected can leave their genetic footprint for longer than average, making them prime targets for detection.

Estimating the integration time makes use of the assumption that both LTRs of a retrovirus are identical at the time of infection. Once the retrovirus lodges itself in the germline, both LTRs evolve separately as if they were paralogs. This is a consequence of the particular replication cycle of the retrovirus, where both LTRs are copied from one and same template during a multistep complex process [13]. By taking into account the 3′LTR-5′LTR sequence divergence and empirical evolutionary rates for some ERV families, researchers have been calculating integration times based on the simple distance over rate formula. However, this method neglects several mechanisms of the ERV loci and the LTRs themselves. First, 3′ LTR and 5′ LTR have different evolutionary rates that depend on selective pressures on each end of the locus. Second, different species may have different evolutionary rates for homologous ERV loci. By using phylogenetic data when available, we hope to surpass these obstacles in obtaining more accurate estimations of integration times.

**Figure 1:**
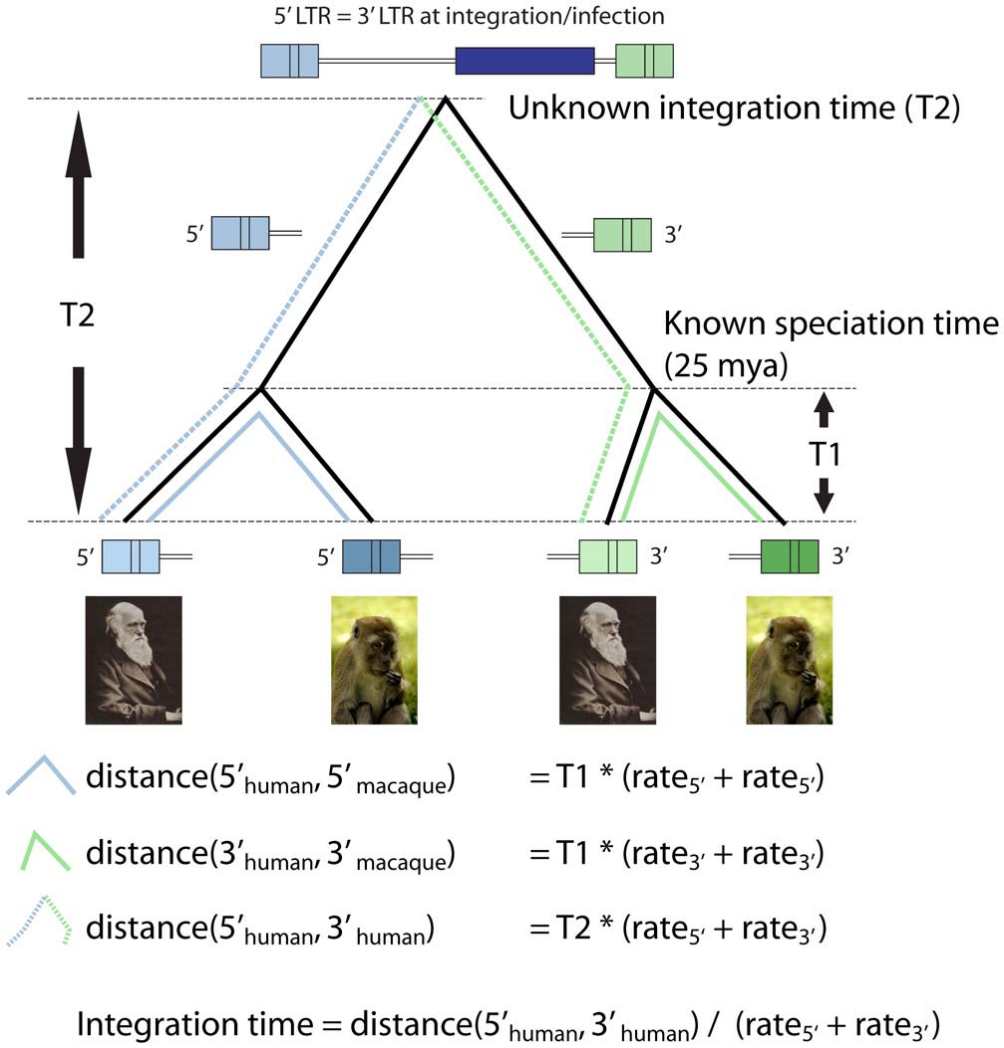
Two species estimation of integration time. Estimation of insertion times based on multi-species phylogenetic data. LTR insertion date can be estimated through phylogenetic data by adding known speciation times of species pairs where those LTRs are known to be present (T1). Calculating the substitution rates for each separate species along T1 (bold blue line, bold green line) and LTR along T2 (dashed lines) as shown in the figure will allow the implementation of a final corrected estimation date (bottom formula).

Assuming known speciation times, we can estimate ERV integration times by using LTR sequence divergence and both 3′ LTR and 5′ LTR rates (figure 1). If we take T1 as the known speciation time, corresponding to the time that each 3′LTR and 5′LTR take to coalesce into their common ancestors, and T2 as the unknown speciation time, for two species A and B we have that Integration time = Distance(5′LTR−3′LTR)/(rate_5′_+rate_3′_). Here, rate_5′_ and rate_3′_ is calculated as the average 5′LTR and 3′LTR evolutionary rates across branches (between species), respectively. By using this approach, we expect to improve the simple fixed rate method with phylogenetic corrections on the estimation.

We also estimated integration times using a computational approach based on markov-chain monte carlo simulations (MCMC) supported by a relaxed molecular clock model with dated tree nodes. This methodology requires a more thorough setup, more detailed in the materials and methods section. By comparing the results obtained from all methods we expect to draw conclusions as to the usefulness and drawbacks of these methods.

## Results

After the initial sequence selection process, we conducted the research on ten endogenous retroviral loci, all with full LTR sequences, present at least in two primate species and whose phylogeny obey the primate evolutionary history.

Using the basic phylogenetic data to infer integration dates, we obtained point estimates of integration time for each endoretroviral sequence. We performed the analysis using both HKY and GTR substitution models when building phylogenies. MCMC estimation of node ages, however, provided us with confidence intervals of integration time (table 1).

**Table 1:** Integration times estimated from independent LTR substitution rates and phylogenies, compared with MCMC estimations.

File	HKY	GTR	MCMC		
			Med	Mean	Int
*ERV3*	42.44	42.13	35.62	36.05	29.52–44.89
*ERVIPF10H*	24.19	24.07	31.77	32.09	27.11–38.84
*ERV PB1*	53.58	54.74	56.58	58.19	39.51–85.82
*ERV WE1*	19.68	19.64	18.27	18.54	14.97–23.76
*ERV FRD*	165.6	166.5	105.8	106.9	96.27–123.2
*ERVK3*	24.49	24.63	32.32	33.11	23.69–47.30
*ERVK9*	58.89	59.11	48.17	48.69	39.18–61.12
*ERVK2*	9.270	9.269	9.131	9.025	7.155–10.37
*ERVK7*	6.373	6.375	7.008	7.065	5.577–8.878
*ERVP4*	38.98	39.43	40.13	40.69	32.44–52.04

Integration time estimates in million years ago (Mya) for the 10 LTR loci using independent LTR rates and several phylogenetic inference methods. For the HKY and GTR substitution models, the genetic distance between LTRs was calculated using the Maximum Composite Likelihood method in MEGA 4.0. MCMC 95% confidence interval of the first node age calculated using a 25000 sample analysis after an initial 50000 sample stabilization run. A sample every 100 from the analysis was taken to build the Bayesian estimation. Three separate runs were made and all values for node ages were congruent (data not shown).

Although the basic phylogenetic method gives us only a point estimate, that specific point in time is, in eight out of ten cases studied, within the confidence intervals obtained by the MCMC calculations. In order to assess how well the use of a single fixed rate of evolution for the entire endoretroviral sequence would fit the MCMC results, we used four estimated rates of evolution and two intervals of rates for human ERVs from the literature, redoing the calculations for integration time with the methodology depicted in figure 1 (table 2).

**Table 2:** Integration times from traditional LTR divergence analysis.

*File*	R = 0.002^(1)^	R = 0.0026^(2)^	R = 0.0014^(3)^	R = 0.0013^(4)^	0.0023<R<0.005^(5)^	0.0025<R<0.0045^(6)^
*ERV3*	26.11	20.08	37.30	40.17	10.44–22.70	11.60–20.89
*ERVIPF10H*	16.85	13.18	24.07	25.93	6.741–14.65	7.490–13.48
*ERV PB1*	22.75	17.50	32.50	35.00	9.101–19.78	10.11–18.20
*ERV WE1*	15.15	11.66	21.65	23.31	6.062–13.18	6.735–12.12
*ERV FRD*	98.32	75.63	140.5	151.3	39.33–85.49	43.70–78.66
*ERVK3*	32.73	25.18	46.76	50.36	13.09–28.46	14.55–26.19
*ERVK9*	39.11	30.09	55.87	60.17	15.65–34.01	17.38–31.14
*ERVK2*	18.49	14.23	26.42	28.45	7.397–16.08	8.219–14.80
*ERVK7*	16.88	12.99	24.12	25.98	6.754–14.68	7.504–13.51
*ERVP4*	32.95	25.35	47.07	50.69	13.18–28.65	14.65–26.36

Integration time estimates (Mya) calculated by using fixed global rates. (1) Andersen *et al* (1997), (2) Lavrentieva *et al* (1998), (3) Lebedev *et al* (2000), (4) Majer and Freeman (1995), (5) Wang *et al* (2007), (6) Johnsson and Coffin (1999). Human 5′-3′ pairwise distances calculated on HKY model phylogenetic trees. Genetic distances calculated in MEGA 4.0 using the maximum composite likelihood model.

**Figure 2:**
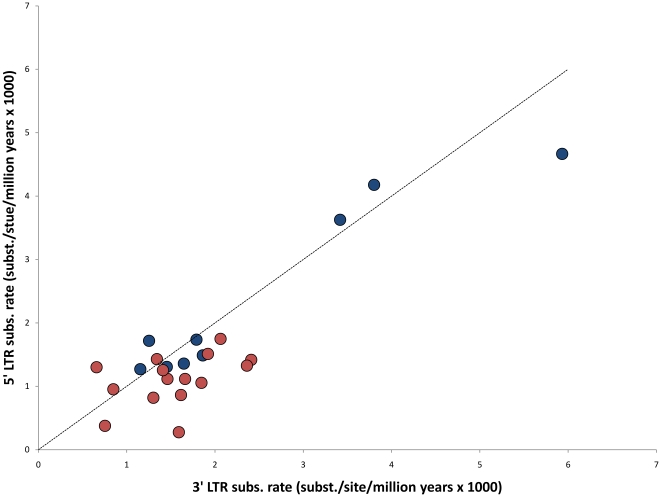
LTR substitution rates. Comparison of substitution rates between 5′ and 3′ LTRs. In blue, pairwise rates for loci estimated to be less than 25 million years old; in red, rates for loci estimated to be more than 25 million years old. The black dashed line represents identical rates between 5′ and 3′ LTRs.

We also estimated 5′ and 3′ LTR substitution rates from different species pairs (table 3) showing a substantial variation in LTR substitution rates between the analyzed ERV families (figure 2). Here, the cutoff point for visual distinction of 25 Mya was used as an approximate date for the New World – Old World Monkey split [14]. This split easily indicates that the LTRs with the faster substitution rates are those of a more recent insertion time, namely the ERVK2, ERVK7 and ERVK9 along the Human-Chimp branch.

**Table 3:** Variation of 3′ LTR and 5′ LTR substitution rates.

	*Homo sapiens/Pan troglodytes*			*Homo sapiens/Pongo abelii*			*Homo sapiens/Macaca mulatta*		
	5′ LTR	3′ LTR	Avg	5′ LTR	3′ LTR	Avg	5′ LTR	3′ LTR	Avg
*ERV3*	0,862	1,615	1,238	0,819	1,303	1,061	1,429	1,342	1,385
*ERVIPF10H*	1,718	1,254	1,486	1,358	1,647	1,502	1,271	1,352	1,311
*ERV PB1*	0,377	0,754	0,565	0,952	0,849	0,901	1,301	0,659	0,980
*ERV WE1*	1,306	1,454	1,380	1,488	1,863	1,675	-	-	-
*ERV FRD*	0,276	1,591	0,933	1,116	1,462	1,289	1,254	1,411	1,332
*ERVK3*	3,626	3,418	3,522	1,736	1,791	1,763	-	-	-
*ERVK9*	1,417	2,411	1,914	1,116	1,662	1,389	1,326	2,362	1,844
*ERVK2*	4,177	3,803	3,990	-	-	-	-	-	-
*ERVK7*	4,665	5,933	5,299	-	-	-	-	-	-
*ERVP4*	1,748	2,063	1,905	1,055	1,847	1,451	1,511	1,920	1,715

Substitution rate estimates along HKY tree branches in number of substitutions per site per 10^3^ million years. Rates are given for each species pair in the tree, calculated from genetic distance between species pairs and assumed speciation times. These rates were used to calculate integration time estimations. For the GTR tree (data not shown), corresponding rates were calculated using the same methodology.

## Discussion

It is clear that, when using a single rate for the whole ERV sequence for integration time calculations, several important factors are being omitted. The final estimation is highly dependent on the original assumption on how fast the endoretroviral sequence is evolving, and for old sequences estimations can vary up to 50% (see Table 2). It was also clear from our study that 5′ and 3′ LTRs have distinct evolutionary rates; that 3′ rates are slightly higher than 5′ rates and that overall rates varies greatly between ERV families (see figure 2 and Table 3). Thus, applying a single evolutionary rate to estimate the time of integration is rarely a good approximation when studying ERV sequences. Point estimates of integration dates are hard to find in the literature, except for the more recent ERV-K group. Most of them are based on fossil records of species separation, namely the New World/Old World monkeys split or the Hominid split. We compared available integration time estimates from the literature with those found by the methods described in this work.

Two works by Zanotto et al [15], [16], estimated an average integration time of the ERV-K group in the human-chimpanzee cluster to 18,3 million years before present (MYBP). That estimate shrunk to 7.8 MYBP, when the analysis was constricted to only the ERV-K present in humans. The latter results are consistent with the findings of our work for the most recent ERV-K loci studied, ERV-K2 and ERV-K7. However, ERV-K loci derived from older lineages, such as the ERV-K3 and ERV-K9, imply a much older integration time. The variability in integration times and rates found within the ERV-K family may discourage a broad generalization on their properties.

Other estimates point out only rough time intervals of estimation. ERV3 is thought to be originated more than 30 million years ago [17], an assumption verified by our results that place the ERV3 integration around 42 million years ago. The ERVPB1 locus had been previously timed around 30 million years old based on PCR amplification from different primates [18], an estimate placing the integration of ERVPB1 at a more recent time than both of the phylogenetic LTR divergence (53,58–54,74 Mya) and the MCMC estimation (39,5–86,3 Mya). ERV-WE1, also known as syncytin-1, is assumed to have infected a Catarrhine ancestor 25–40 million years ago [19], although our study reveals a somewhat more recent integration. This is explained by the fact that LTR position for the rhesus macaque ERV-WE1 locus was coincident with a gap in genomic data (Jan. 2006 assembly) and therefore, we couldn't include it in our study. ERV-FRD, also known as syncytin-2, is thought to be much older, over 40 million years [20], and our results support that this endoretroviral integration is quite ancient. The estimated integration time of over 100 million years would suggest that a homologous ERV-FRD could be found on small mammals, but this is not the case. Even though mice possess their own syncytins, syncytin-A and syncytin-B, these do not share a common ancestor with the primate syncytins [21]. The old age of syncytin-2 may also be explained by an induced bias due to the fact that this locus has a slower evolutionary rate than any other studied ERV.

Comparing the basic phylogenetic method with the more computationally intensive MCMC method, we find that for recent (<40 million years) ERVs the predictions of both methods are quite similar. Problems arise for old (>40 million years) ERVs, where the predictions of the phylogenetic method tend to diverge from those of the MCMC with the increasing ERV age. The use of a fixed rate, however, produces results that vary considerably with ERV family and the actual evolutionary rate of the sequence, and should be avoided whenever multi-species phylogenetic data are available. The MCMC calculations can be quite consuming if a big amount of data is needed. For genome-wide studies, the simple phylogenetic approach may constitute a viable and faster alternative, while maintaining a certain level of accuracy.

## Methods

### Sequence mining

We selected several known human endogenous retroviruses and acquired their sequences from the UCSC Genome Browser [22], with the aid of a custom track designed to help the visualization of endoretroviral sequences. A DNA dot plot [23] was used to confirm the presence of long terminal repeats (LTRs). Human endogenous retroviral sequences were used as a template to detect homologous endoretroviral sequences in other primates, whenever possible. We cropped the LTRs and aligned them using the Clustal algorithm [24]. We conducted a phylogenetic analysis of every endoretroviral sequence by building phylogenetic trees of both 3′ and 5′ LTRs under several models. Endoretroviral sequences that presented strange behavior, such as a marked non-grouping of 3′ and 5′ LTRs into monophyletic groups or mismatches with known primate evolution, were disregarded from posterior analysis. Following is a list of the included sequences and their accession numbers with human genome coordinates: ERV3 [NT_007933.15; Chr7:64,450,201–64,460,983], ERVIPF10H [NT_010194.17; Chr15:80,207,780–80,213,351], ERV-PB1 [NT_026437.12; Chr14:93,085,828–93,096,468], ERV-WE1 [NT 007933.15; Chr7:92,086,915–92,117,832], ERV-FRD [NT 007592.15; Chr6:11,102,722–11,111,959], ERVK3 [NT 011295.11; Chr19:11,824,892–11,833,002], ERVK9 [NT 011295.11; Chr19:9,425,141–9,435,002], ERVK2 [NT 077531.4; Chr8:8,092,084–8,101,696], ERVK7 [NT 009237.18; Chr11:3,425,232–3,434,785] and ERVP4 [NT 010966.14; Chr18:31,663,560–31,675,827]. Full dataset accession numbers and original sequence lengths can be consulted in supplementary table S1.

### Phylogenetic analysis

Phylogenetic trees for the LTRs were built using the PhyML software, using HKY+G and GTR+G substitution models [25] under the maximum likelihood method, with default parameter values and 1000 bootstrap replicates. We used the mcmctree application in the PAML software package [26] to estimate node ages in the HKY trees, using a non-informative prior and empirical species split time intervals for calibrating the molecular clock model. Branch lengths were automatically extracted from the tree files using the newick tools 0.1 software package [27]. Nucleotide distance was calculated in MEGA 4.0 [28] using the maximum composite likelihood model.

### Time of integration estimation

In order to estimate time of integration from basic phylogenetic data, we used the method described in figure 1. Independent 3′ and 5′ rates were estimated using the maximum composite likelihood model with gamma distributed rates among sites in MEGA 4.0. The gamma shape parameter was estimated for each dataset using the jModelTest substitution model selection functionality. Applying the values for the estimated genetic distances (D) and known speciation times (T) in the formula D = T*2k, where k is the substitution rate, a rate of substitutions per site per year can be inferred. This method is applied in all sequence pairs of each dataset as shown in figure 1 in order to obtain a final value for the integration time.

The evolutionary rate of the ancestral ERV sequence, corresponding to the branch prior to the last species node, was considered to be, as a simplification, an average of the evolutionary rates across the remaining branches. The HKY+G model was selected after a model fit analysis using the corrected Akaike Information Criteria test. Two of the datasets yielded the K80+G model as the best fit but, since the HKY+G model came in close second in both those cases, the latter model as used throughout the analysis in order to allow for a common framework. The GTR+G model was also included as the next best fit for all the datasets and to serve as a test for congruency of estimations when using a different well fitted substitution model.The MCMC estimation of node ages was performed with mcmctree. Each dataset's phylogeny was assumed to behave as a molecular clock system. Internal nodes of each phylogenetic tree were calibrated with confidence intervals pertaining to speciacion events - the time intervals used were of 4–6 Mya for the human-chimpanzee node, 12–15 Mya for the human-orangutan node and 23–27 Mya for the human-rhesus macaque node. Node age estimation was performed in triplicate for each sequence set to validate results. Each MCMC chain ran for 75000 steps of which the last 25000 contributed with 250 samples for the estimation.

## Supporting Information

Table S1 Supplementary table 1 lists all 10 datasets for each mammalian host used in our study, along with accession numbers and sequence lengths for all genomic sequences used.
(0.05 MB DOC)
