# Supplementary material for: Improved Integration Time Estimation of Endogenous Retroviruses with Phylogenetic Data
Source: PLoS One. 2011 Mar 4;6(3):e14745. doi: 10.1371/journal.pone.0014745 (PMC3048862; doi:10.1371/journal.pone.0014745)
Supplement: Table S1 — Supplementary table 1 lists all 10 datasets for each mammalian host used in our study, along with accession numbers and sequence lengths for all genomic sequences used. (0.05 MB DOC) [file pone.0014745.s001.doc]

**Supplementary table S1 – list of all 10 datasets for each mammalian host used in our study, along with accession numbers and sequence lengths for all genomic sequences used.**

| ERV locus | Mammalian host | Accession number | Length (bp) |
| --- | --- | --- | --- |
| *ERV3* | *Homo sapiens* | NT 007933.15 | 10782 |
|  | *Pan troglodytes* | AC 192481.3 | 10983 |
|  | *Pongo abelii* | AC 200427.2 | 10931 |
|  | *Macaca mulatta* | AC 210126.3 | 11260 |
| *ERVIPF10H* | *Homo sapiens* | NT 010194.17 | 5571 |
|  | *Pan troglodytes* | AC 159217.3 | 5738 |
|  | *Pongo abelii* | AC 207004.4 | 5831 |
|  | *Macaca mulatta* | AC 200687.3 | 5719 |
| *ERV PB1* | *Homo sapiens* | NT 026437.12 | 10640 |
|  | *Pan troglodytes* | AC 194649.3 | 10643 |
|  | *Pongo abelii* | AC 206925.4 | 10716 |
|  | *Macaca mulatta* | AC 148685.1 | 10296 |
| *ERV WE1* | *Homo sapiens* | NT 007933.15 | 30917 |
|  | *Pan troglodytes* | AC 183672.2 | 30302 |
|  | *Pongo abelii* | AC 199834.2 | 31227 |
| *ERV FRD* | *Homo sapiens* | NT 007592.15 | 9237 |
|  | *Pan troglodytes* | AC 182639.3 | 9225 |
|  | *Pongo abelii* | AC 200331.2 | 9223 |
|  | *Macaca mulatta* | AC 193044.4 | 9231 |
| *ERVK3* | *Homo sapiens* | NT 011295.11 | 8110 |
|  | *Pan troglodytes* | AC 193186.3 | 8255 |
|  | *Pongo abelii* | AC 206006.3 | 7898 |
| *ERVK9* | *Homo sapiens* | NT 011295.11 | 9861 |
|  | *Pan troglodytes* | AC 142300.1 | 9853 |
|  | *Pongo abelii* | AC 211912.3 | 9797 |
|  | *Macaca mulatta* | AC 197939.3 | 9980 |
| *ERVK2* | *Homo sapiens* | NT 077531.4 | 9612 |
|  | *Pan troglodytes* | AC 183110.3 | 9190 |
| *ERVK7* | *Homo sapiens* | NT 009237.18 | 9553 |
|  | *Pan troglodytes* | AC 183110.3 | 9576 |
| *ERVP4* | *Homo sapiens* | NT 010966.14 | 12267 |
|  | *Pan troglodytes* | AC 146247.3 | 13041 |
|  | *Pongo abelii* | AC 205931.4 | 12431 |
|  | *Macaca mulatta* | AC 190302.5 | 12234 |
